# *In silico* pharmacological analysis of *Tinospora cordifolia* compounds targeting African swine fever virus B175L

**DOI:** 10.1371/journal.pone.0341938

**Published:** 2026-06-03

**Authors:** Kasuni Karunarathne, Saumya Poorni, Nethra Jayampathi, Hasitha Dhananjaya, Anju Hasintha, Malinda Hulugalla, Thilini A.N. Mahakapuge, Nadeeka Nethmini, Naveed Iqbal, Barana Jayawardana, Lakmal Ranathunga

**Affiliations:** 1 Department of Animal Science, Faculty of Agriculture, University of Peradeniya, Peradeniya, Sri Lanka; 2 Faculty of Veterinary Medicine and Animal Science, University of Peradeniya, Peradeniya, Sri Lanka; 3 School of Dentistry and Medical Science, Faculty of Science and Health, Charles Sturt University, Wagga Wagga, NSW, Australia; 4 School of Interdisciplinary Engineering and Sciences, National University of Sciences and Technology, Islamabad, Pakistan; Universidad El Bosque, COLOMBIA

## Abstract

African swine fever virus (ASFV) is a highly lethal DNA virus that suppresses the host’s immune response by establishing infection. B175L, one of its key immune-evasion proteins, directly inhibits STING-mediated type I interferon (IFN-I) signalling, thereby preventing the activation of antiviral defences. Thus, targeting B175L could be a promising strategy for antiviral drug development as effective ASFV inhibitors remain unidentified. In this study, we investigated the potential of *Tinospora cordifolia*’s bioactive compounds to disrupt B175L’s function and restore immune signalling. Gas chromatography-mass spectrometry (GC-MS) analysis of the methanol extract from *T. cordifolia* stems identified 86 compounds. These were filtered using SwissADME, ProTox 3.0, and DataWarrior, yielding 15 compounds with favourable drug-likeness and safety profiles. We generated a highly accurate 3D model of ASFV B175L with strong confidence scores for the structural accuracy, using AlphaFold3. The filtered compounds were then subjected to virtual screening with PyRx 0.8, and the 3 compounds with binding affinities ≤ −6 kcal/mol were selected for subsequent analysis. Molecular dynamics (MD) simulations were used to assess binding stability, including root mean square deviation and fluctuation (RMSD, RMSF), protein-ligand contacts, radius of gyration (rGyr), and solvent accessible surface area (SASA) using Schrödinger Maestro. Taken together, these *in silico* results suggest that *T. cordifolia-*derived Benzaldehyde, 5-bromo-2-hydroxy-, (5-trifluoromethyl-2-pyridyl) hydrazone, Carbamic acid, N-(3-oxo-4-isoxazolidinyl)-, benzyl ester, and 1H-Indol-5-ol may act as potential inhibitors of B175L and represent preliminary antiviral hits against ASFV, warranting further *in vitro* and *in vivo* validation.

## Introduction

African swine fever (ASF) is a highly lethal and contagious viral disease that affects both domestic and wild pigs. Acute infections are often fatal, with a mortality rate approaching 100%. ASF is classified as a notifiable disease by the World Organisation for Animal Health (WOAH) due to its severe economic and animal health impact [1]. The disease was first documented in Kenya in 1921 and has since become a global concern. The current transboundary epidemic began with the introduction of the highly virulent ASF virus (ASFV) genotype II into Georgia in 2007 [2]. As of November 2024, ASF has been reported in 63 countries and territories spanning five major regions. Between January 2022 and November 2024, the disease affected over 781,000 domestic pigs and 27,400 wild boars, leading to the loss of more than 1.87 million animals [3]. ASFV belongs to the genus *Asfivirus* within the family *Asfarviridae*. It is a large, complex, double-stranded DNA virus with genome sizes ranging from 170 to 194 kilobases (kb) among different isolates. The viral genome encodes approximately 150–200 proteins that perform diverse functions, including viral structure formation, replication, and immune evasion [4].

The lack of commercially approved vaccines or effective therapeutics for ASF has escalated into a major crisis for the global pork industry [5]. Although live-attenuated vaccines (LAVs) have shown potential, their use is limited by safety concerns, including the risk of reversion to virulence and the possibility of persistent infections. Subunit vaccines, while generally safer, still require further investigation to identify effective antigens and suitable adjuvants that elicit strong, durable immune responses [6]. In addition to these scientific hurdles, vaccine development faces practical challenges, including adverse side effects, limited diagnostic capabilities, insufficient data for risk assessments, biological complexity, and high research costs [7]. As a result, there is an urgent need to explore alternative strategies for ASF control, particularly through the discovery of safe and effective antiviral agents [8]. Natural plant-derived compounds offer a promising alternative for antiviral drug discovery due to their vast chemical diversity and established antiviral activity. Numerous medicinal plants have demonstrated efficacy in inhibiting viral infections, and extensive research supports the antiviral activity of plant-derived bioactive compounds in both *in vitro* and *in vivo* models [2]. In this context, computer-aided drug design (CADD), also known as *in silico* drug discovery, has emerged as a powerful tool in modern pharmacological research. These computational approaches are not only cost-effective and time-efficient but also minimise the reliance on animal testing in early-stage drug development [9].

**Fig 1:**
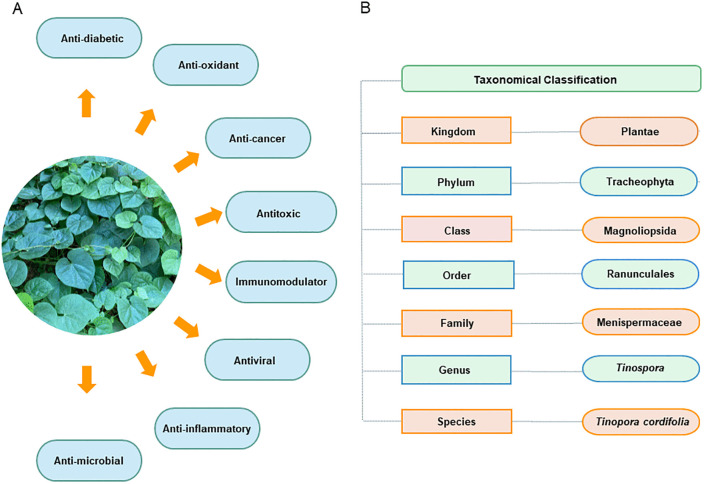
Overview of *Tinospora cordifolia.* (A) Reported pharmacological activities of *Tinospora cordifolia*, including anti-diabetic, anti-oxidant, anti-cancer, antitoxic, immunomodulatory, antiviral, anti-inflammatory and anti-microbial effects. (B) Taxonomic classification of *T. cordifolia*.

*Tinospora cordifolia* is a traditional medicinal plant widely used in Ayurveda for its well-documented broad range of pharmacological activities (Fig 1A) [10,11] mediated through a diverse repertoire of alkaloids, glycosides, diterpenoid lactones, and polysaccharides [11,12]. Taxonomically, it belongs to the kingdom Plantae, phylum Tracheophyta, class Magnoliopsida, order Ranunculales, family Menispermaceae and genus *Tinospora* (Fig 1B). It is distributed throughout the tropical regions, especially in India and Southeast Asia, and is also endemic to Sri Lanka [11]. In recent years, *T. cordifolia* has been extensively investigated for its potential in the management of viral diseases, with growing evidence supporting its antiviral efficacy. Clinical and experimental data indicate that *T. cordifolia* exhibits antiviral activity against several viral infections, including DNA viruses such as herpes simplex virus type 1 (HSV‑1), and RNA viruses including human immunodeficiency virus (HIV), hepatitis A virus (HAV), chikungunya virus (HIKV) and severe acute respiratory syndrome coronavirus 2 (SARS-CoV-2) [13–16].

Among the many proteins encoded by ASFV, B175L has recently been identified as a key immunomodulatory factor [17]. It was reported that B175L plays a critical role in suppressing the type I interferon (IFN) response, a central antiviral defence mechanism. Specifically, B175L inhibits IFN-β production and downstream signalling by directly targeting STING (Stimulator of Interferon Genes) and its ligand 2′3′-cyclic GMP-AMP (2′3′-cGAMP), both essential components of the cyclic GMP-AMP synthase-STING (cGAS-STING) pathway. This interaction disrupts the activation of transcription factors IRF3 and NF-κB, ultimately suppressing IFN-stimulated gene expression. Due to its strong immune-suppressive function, ASFV B175L represents a promising therapeutic target for antiviral intervention.

Given that the ASFV B175L protein functions as a key negative regulator of the host’s antiviral interferon response through its interaction with STING, this *in silico* study aims to identify ligands derived from *T. cordifolia* that can effectively bind to B175L. By disrupting the B175L-STING interaction, the study seeks to restore STING-mediated innate immune signalling. This approach presents a novel and promising strategy for developing antiviral therapeutics against ASF.

## Materials and methods

### Overview of the study workflow

**Fig 2:**
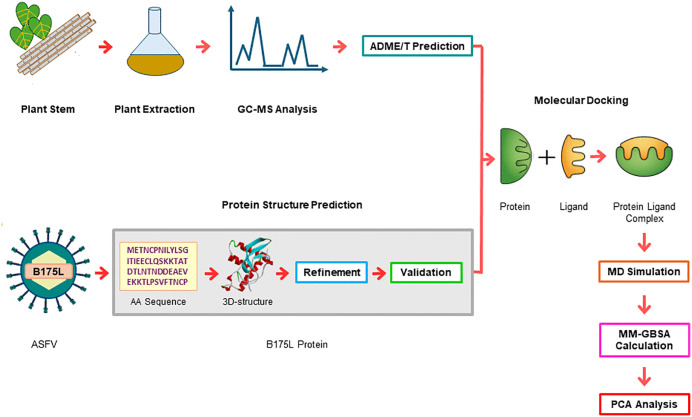
Workflow of the study.

A schematic representation of the study workflow is shown in Fig 2. The study followed an integrated workflow encompassing the preparation of selected plant extracts, phytochemical identification by GC-MS analysis, and a bioinformatics pipeline to identify promising phytochemicals for inhibition of ASFV targeting the B175L protein. In the *in silico* workflow, phytochemicals identified by GC-MS that met predefined ADME/T criteria were selected for virtual screening against the protein, whose structure was generated via deep-learning-based structure prediction, and subsequently refined, and validated. The top-ranked complexes were further investigated using MD simulations, MM-GBSA calculations, and PCA of the trajectories.

The schematic summarises the overall design of the study, including sequential integration of experimental phytochemical extraction with computational modelling. ASFV: African swine fever virus; GC-MS: gas chromatography-mass spectrometry; ADME/T: absorption, distribution, metabolism, excretion, and toxicity; MD: molecular dynamics; MM-GBSA: molecular mechanics/generalised born model and solvent accessibility; PCA: principal component analysis. Red arrows indicate the directional flow of the methodology from initial sample preparation to final trajectory analysis.

### Plant material collection, authentication, and preparation

In this study, the stems of *Tinospora cordifolia* were systematically collected in December 2024 from a privately-owned herb garden located in the Rathnapura district, Sri Lanka, and identified and authenticated by a botanist [18,19]. Upon arrival at the laboratory, we thoroughly cleaned the herb stems to remove dirt and debris. The stems were cut into small pieces, sun-dried, and ground into a powder using an electric grinder. The powder obtained was sieved (0.2 mm) using a stainless-steel sieve and stored in an airtight polythene bag at −20 °C for further analysis [20].

### Plant extraction preparation

We weighed 7.5 g of the sample for extraction using an analytical balance. The sample was mixed with 250 mL of ≥99.9% methanol (VWR CHEMICALS; 85681.320) using a Sonicator (Model CL-188, U.S.A) for 15 minutes at 27 °C at 150 rpm. The mixture was filtered with Whatman No. 01 paper. Then, the supernatant was evaporated at 40 °C using a rotary evaporator under vacuum conditions. The resulting pulp-like residue was collected, and 1 mg of the residual was transferred into a 2 mL Eppendorf tube and dissolved in 1 mL of methanol for GC-MS analysis [20,21].

### Gas chromatography-mass spectrometry analysis

Gas chromatography-mass spectrometry (GC-MS) is a rapid and sensitive technique for identifying key bioactive constituents in complex plant extracts [22]. Phytochemical analysis of the methanolic stem extract of *T. cordifolia* was carried out using a GC-MS analyser (Agilent Technologies 7890B GC system) coupled to a 5977A mass-selective detector (MSD) and a 7890B autosampler. An Agilent DB FATWAX UI column (30 m × 0.250 mm i.d. × 0.25 µm film thickness) was used, with helium as the carrier gas at a constant flow rate of 1.5 mL/min. Samples were analysed by injecting 1 µL of extract in split mode (split ratio 2:1) into an inlet maintained at 250 °C. The oven temperature programme was as follows: initial temperature 40 °C ramped at 5 °C/minute to 115 °C held for 5 minutes, then increased at 5 °C/minute to 140 °C and held for 5 minutes, followed by a ramp of 2 °C/min increase to 210 °C and held for 11 minutes and, finally increased at 5 °C/minute to 240 °C held for 9 minutes. The total run time lasted for 91 minutes. Before injection, samples were filtered through PTFE membrane filters, and the filtrate was injected directly. Phytochemical identification was performed by comparing the mass spectra of the detected peaks with those in the national institute of standard and technology 2014 (NIST14) and wiley registry 9^th^ edition/NIST 2011 (W9N11) mass spectral libraries [23]. Peak-by-peak deconvolution and library matching were performed using GC-MS ChemStation software. Only compounds with a library match probability score of at least 50% were considered for tentative identification [24,25]. Putative identities obtained from library matching were further verified using the PubChem database (https://pubchem.ncbi.nlm.nih.gov/), and the corresponding compound information and three-dimensional (3D) structures were downloaded from the PubChem database for subsequent analyses in the study.

### ADME/T prediction

To evaluate the *in silico* ADME/T (absorption, distribution, metabolism, excretion, and toxicity) properties of the plant compounds, we used SwissADME (http://www.swissadme.ch/), DataWarrior (https://openmolecules.org/datawarrior/download.html), and ProTox 3.0 (https://tox.charite.de/protox3/) [26]. SwissADME is an online tool that predicts pharmacokinetic properties. The SMILES of the compounds were obtained from the PubChem database and inserted into the SwissADME interface [27]. ADME properties were evaluated based on pharmacokinetic properties such as physicochemical properties, lipophilicity, water solubility, pharmacokinetic, drug-likeness, and medicinal chemistry of plant compounds [28]. Physicochemical properties included molecular weight, number of heavy atoms, number of aromatic heavy atoms, number of rotatable bonds, and H-bond acceptors/donors, which define molecular size, flexibility, polarity, and hydrogen-bonding capacity, all of which directly affect membrane permeability and oral absorption potential. Lipophilicity was examined via the Moriguchi LogP (MLOGP). This parameter predicts passive permeability compound absorption. Water solubility was estimated using the ESOL model (Log S). Drug candidates must be sufficiently soluble to enable absorption with reasonable solubility for oral dosage forms. Pharmacokinetics assessments focused on gastrointestinal absorption, both predicted and measured. High predicted gastrointestinal (GI) absorption is considered as a favourable property for oral candidates, as it suggests a potential for efficient bioavailability *in vivo.* Drug likeness was filtered using Lipinski’s Rule of Five (molecular weight ≤500 Da, H bond acceptors ≤5, H bond donors ≤5, and MLOG ≤4.15) and Bioavailability score. In Lipinski’s Rule of Five, compounds with no more than two violations were selected, as multiple violations are empirically linked to poor oral bioavailability in drug development [29,30]. In bioavailability scores, ≥ 0.55 is statistically more likely to achieve at least 10% oral bioavailability, which is crucial for early-stage selection. We assessed the medicinal chemistry of compounds using synthetic accessibility scores; lower scores indicate easier synthesis for drug development.

Safety of the compounds was evaluated using the ProTox 3.0 web server and DataWarrior software [31]. Canonical SMILES of each compound were employed to assess critical toxicological endpoints. ProTox 3.0 provides comprehensive toxicity evaluation by predicting endpoints such as acute oral toxicity (rat LD50 value), cardiotoxicity (caused by human ether-à-go-go-related gene (hERG) channel. This potassium channel plays a crucial role in regulating the cardiac action potential), AMES mutagenicity, carcinogenicity, cytotoxicity, immunotoxicity, and hepatotoxicity. These predictions offer insight into the compound’s potential impact on various organ-specific and pathway-based toxicities [32]. DataWarrior software was employed to predict mutagenicity, irritancy, tumourigenesis, and reproductive toxicity, using structural alerts associated with established safety parameters for selected ligand compounds [33,34].

### Protein sequence retrieval and characterisation

The amino acid sequence for the protein B175L from the ASFV Georgia strain was obtained from the UniProt database (UniProt ID: A0A2X0RVG4) (https://www.uniprot.org/) [35,36]. Basic chemical and physical properties of ASFV B175L, such as molecular weight, amino acid composition, theoretical pI, instability index, extinction coefficient, atomic composition, estimated half-life, the total number of positively charged residues, the total number of negatively charged residues, aliphatic index, and grand average of hydropathic were analysed using ExPASy ProtParam tool (https://www.expasy.org/) to assess protein stability, solubility, and suitability of the protein for subsequent docking based inhibitor screening [37–39].

### Protein structure prediction and refinement

The 3D structure of the protein was predicted using AlphaFold3 (https://alphafoldserver.com/), an advanced deep-learning-based biomolecular structure prediction tool [39,40]. The prediction was performed by feeding the primary amino acid sequence into the AlphaFold server. The quality of the predicted model was assessed using structural confidence metrics, including the predicted local distance difference test (pLDDT), predicted aligned error (PAE), predicted template modelling (PTM) score, and interchain predicted TM-score (ipTM) [40]. To improve the quality of the predicted model, such as folding accuracy, structural stability, and minimisation of steric clashes, the 3D structure was refined using GalaxyRefine (https://galaxy.seoklab.org/cgi-bin/submit.cgi?type=REFINE) [41]. In protein structure prediction, structure validation is a vital step. The basic conformation of the suggested protein model was obtained by submitting it to ProSA-web (https://prosa.services.came.sbg.ac.at/prosa.php) [42].

### Protein structure validation

The predicted and refined 3D structure was evaluated using the SAVES 6.0 (https://saves.mbi.ucla.edu/) structure evaluation server as a common platform for quality checks, error assessment, and model suitability for further research, utilising 3 assessments: PROCHECK stereochemical assessment, Verify3D, and ERRAT quality factor, and the Ramachandran plot. PROCHECK visualised the backbone dihedral angles ψ against φ of amino acids in the protein structure [43, 44]. Verify3D assessed the compatibility of a 3D atomic model with its corresponding 1D amino acid sequence [42]. To further validate the protein model, MolProbity (https://molprobity.biochem.duke.edu/) was used, which provides a log-weighted score combining the clash score, the percentage of Ramachandran outliers, and the percentage of unfavourable side-chain rotamers [45]. Final assessment of model structural quality was performed using ProSA-web (https://prosa.services.came.sbg.ac.at/prosa.php), which evaluates the overall model quality by comparing its energy distribution to those of experimentally determined Protein Data Bank structures [42, 46].

### Molecular docking and visualisation

Molecular docking techniques were performed to select more potent and efficient ligand compounds capable of interacting with the binding pocket of the target protein [47]. Ligands and target protein docking were performed using the Autodock Vina function of PyRx software version 0.8 (https://sourceforge.net/projects/pyrx/files/latest/download), and docking poses were visualised and analysed using BIOVIA Discovery Studio 2024 (https://discover.3ds.com/discovery-studio-visualizer-download).

For molecular docking, the protein structure was prepared using BIOVIA Discovery Studio 2024, followed by energy minimisation with SWISS PDB Viewer (https://spdbv.unil.ch/), which facilitated structural stabilisation. It corrects distorted geometry by adjusting atomic positions to relieve internal strain and thereby lower the protein’s overall energy [48]. The 3D SDF conformations of all ADME/T-approved plant compounds were downloaded from PubChem [49,50]. All ligands underwent geometry optimisation and energy minimisation using the universal force field (UFF) implemented in Open Babel via the PyRx platform. In PyRx 0.8, the prepared protein and ligands were converted from PDB files to docking-compatible PDBQT format, which includes the adding hydrogens, assigning partial charges, and defining torsion degrees of freedom. A 3D affinity grid box was established with dimensions of X: 86.3617, Y: 69.9772, and Z: 56.0403 Å with a grid spacing of 1.0 Å to ensure sufficient coverage for the binding region. The grid box centre was set to coordinates of X: 10.3647 Å, Y: 2.6009 Å, and Z: 2.3004 Å, and the exhaustiveness parameter for AutoDock Vina was set to 8 [51,52]. These dimensions were carefully chosen to cover entire protein surface, facilitating blind docking strategy. This allowed ligand to search potential binding site across protein surface rather than restricted to a defined active site. For each ligand, the top 10 docking poses were generated, and the pose with the lowest predicted binding energy (kcal/mol) was retained as the representative binding mode [53].

To verify the initial findings, docking scores were independently evaluated using the CB-Dock2 server, which combines curvature-based cavity detection with AutoDock Vina-based blind docking procedure and template-guided docking [54,55]. This improve binding site identification and pose prediction. This secondary screening identified putative binding cavities from protein surface, providing an independent cavity-based assessment of the most probable binding regions and complementing the initial whole-protein blind docking approach. For each ligand-protein pair, CB-Dock2 automatically preprocessed the inputs by adding missing side chains, hydrogen atoms to the protein, removing crystallographic water molecules and adding partial charges. The server carried out docking within the identified top-ranked cavities and corresponding binding energies [55,56]. The complex was visualised using BIOVIA Discovery Studio to characterise interaction types and distances [57,58]. While the estimated docking score was the primary quantitative filter, the selection of the representative binding mode was not entirely based on this energetic ranking. To ensure biological relevance, the highest-ranking poses were also visually inspected to support the plausibility of binding orientation and the formation of interactions with key functional residues. Specific attention was paid to the interactions within and close to the critical MYM-type motif of zinc fingers. The functional domain of B175L was identified using the Conserved Domain Database (CDD) at NCBI (https://www.ncbi.nlm.nih.gov/Structure/cdd/wrpsb.cgi) [59]. This tool identifies conserved protein motifs and domains, clarifying protein functional sites [60].

### Molecular dynamics simulations

The molecular dynamics (MD) simulations were conducted using the Desmond module of the Schrödinger suite to investigate the binding stability of selected protein-ligand complexes [61]. The system was prepared using the System Builder tool, with the simple point charge (SPC) water model as the solvent, in an orthorhombic box with periodic boundary conditions (PBC). To neutralise the system, the counterions, Na^+^ and Cl^-^, were added. The OPLS5e force field was used for energy minimisation and MD simulations. The simulation trajectory spans 100 ns, recording 100 ps intervals, under NPT ensemble conditions at a fixed pressure of 1.01 bar and a temperature of 310 K. The total charge of the protein during the simulations was −5. To analyse structural stability and equilibration behaviour of the simulated complexes, root mean square deviation (RMSD) concerning protein and ligand, root mean square fluctuation (RMSF) to characterise local changes in protein and ligand, detailed protein-ligand contact analyses (including hydrogen bonds, hydrophobic, ionic, and water bridge interactions), secondary structure element (SSE) analyses and prediction of ligand properties such as ligand RMSD, radius of gyration (rGyr), molecular surface area (MolSA), and solvent accessible surface area (SASA), like key structural and dynamic parameters were computed [62].

### Binding energy analyses

The binding free energies (Gbind) of the protein-ligand complexes were generally evaluated using molecular mechanics during MD simulations of the ASFV B175L complexed with ligands, using the molecular mechanics/generalised born model and solvent accessibility (MM-GBSA) module in Prime. The post-docking rescoring approach evaluates the stability of the ligand-protein complexes after binding and can improve docking results by reducing false positives [63]. The OPLS5 force field, VSGB solvent model, and rotamer search methods were applied to compute binding free energies. Following the MD simulation, frames from the MD trajectory were extracted. Overall binding free energies of protein-ligand complexes were calculated using the following equation.


$$\begin{array}{c} \Delta \text{Gbind}=\text{Gcomplex}-(\text{Gprotein}+\text{Gligand}) \end{array}$$

Here, ΔGbind denotes the binding free energy, Gcomplex represents the free energy of the complex, Gprotein denotes the free energy of the target protein, and Gligand corresponds to the free energy of the ligand. Within the MM-GBSA framework, an increasingly negative MM-GBSA binding free energy corresponds to a more favourable and stronger protein-ligand association [64].

### Principal component analyses

Principal component analysis (PCA) is a multidimensional statistical analysis to characterise dominant conformational fluctuations of the protein-ligand complex. In this study, PCA calculations and subsequent visualisation were performed with the Bio3D package in R over the 100 ns of the MD trajectory. A comprehensive comparison of structural and energetic parameters can be used to characterise and differentiate various protein-ligand complexes. These parameters collectively reflect the stability, geometry, and interaction strength within each system, thereby offering deeper insight into their structural and energetic distinctions [65].

## Results

### Plant stem extract profiling via GC-MS

GC-MS analysis revealed the presence of 86 bioactive compounds in the methanolic stem extract of *T. cordifolia*. The GC-MS chromatogram illustrates the separation of these constituents (S1A Fig). The detected compounds, together with their retention time (RT), area %, PubChem ID, molecular formula, IUPAC name, chemical class, and functional groups, are listed in S1 Table. The RT and peak area values ranged from 3.062 to 88.642 minutes and from 0.01% to 26.78%, respectively. The RT distribution reflects differences in physicochemical properties such as polarity, molecular size, and interactions with the stationary phase, whereas peak area % provides a relative measure of compound abundance in the extract [66]. The wide range of RTs and peak areas indicates a chemically heterogeneous mixture with constituents present in varying levels.

The *T. cordifolia* stem methanol extract displayed a diverse chemical profile (S1 Table). Fatty acids and esters (FAMEs/FAEEs), including saturated fatty acids, MUFAs, PUFAs, and fatty acid methyl esters (FAMEs), approximately 27% of the total identified compounds. Ethers and polyethylene glycols (long-chain aliphatic ethers, polyethers, ethoxylated alcohols) comprised about 19%, while macrocyclic compounds and crown ethers (including crown ethers, bis-crown ethers, macrocycles) represented roughly 8%. Alcohols and phenols, encompassing diterpene alcohols, fatty alcohols, phenols, furanones, contributed around 14%. Nitrogen-containing compounds (alkaloids, amines, and nitriles) accounted for approximately 12%. Hydrocarbons (including indole alkaloids, piperidines, pyridine derivatives, aromatic nitriles) represented about 9%, terpenoids and steroids (triterpenes, polycyclic sesquiterpenoids) about 2%, and halogenated and miscellaneous organics (halogenated esters, halogenated thioethers, sulfite esters, silylated derivatives) about 9%.

### *In silico* drug-likeness, pharmacokinetic screening, and toxicity prediction

The GC-MS identified phytochemicals in the plant extract, which were then subjected to comprehensive ADME profiling using SwissADME. Drug-likeness was assessed with respect to Lipinski’s rule of five. As shown in the S2 Table, 5 compounds violated Lipinski’s rule, while 31 compounds showed lower GI absorption. The majority of the compounds, including those with violations, met the oral bioavailability (≥0.55), a lower number of rotatable bonds (≤9), a preferable synthetic accessible score (≤6), ensuring favourable oral absorption, molecular flexibility, and optimising pharmacokinetic viability subsequently in a typical drug development context [67].

Then, the toxicity risk assessment was performed using a combination of endpoints from ProTox 3.0 and DataWarrior. As indicated in the S2 Table, 10 compounds were classified below toxicity class III, indicating low toxicity. In contrast, the majority of the ligand compounds were in low-toxicity classes with higher lethal doses (LD50 in rats) compatible with safe exposure [68]. Almost all the compounds were positive for very few toxicity criteria. Further evaluation of endpoints such as mutagenicity, tumourigenicity, reproductive toxicity, and irritant was conducted using DataWarrior software, and 43 ligands were predicted to be negative for these endpoints. Based on comprehensive ADME and toxicity assessments, 15 compounds were selected for further analysis (Tables 1 and 2).

**Table 1:** Predicted pharmacokinetic properties of selected compounds using SwissADME.

S. No	PubChem ID	Heavy Atoms	Aromatic Heavy Atoms	Rotatable Bonds	Total Molweight (g/mol)	MLOGP	H-Acceptors	H-Donors	Lipinski (Yes/No)	Bioavailability Score	Log S (ESOL)	GI Absorption	Synthetic Accessibility
L3	136651250	21	12	4	360.132	3.01	6	2	0/Yes	0.55	−4.76	High	2.81
L4	337121	7	0	2	92.1405	3.62	0	0	0/Yes	0.55	−1.49	Low	3.12
L6	561323	17	6	5	236.226	0.35	4	2	0/Yes	0.55	−1.64	High	3.26
L9	16054	10	9	0	133.15	0.91	1	2	0/Yes	0.55	−1.98	High	1
L13	9015	9	6	1	124.139	1.15	2	1	0/Yes	0.55	−2.03	High	1
L23	119838	10	0	0	144.126	−1.77	4	2	0/Yes	0.85	−0.5	High	3.6
L24	70627	10	6	0	142.11	−1.42	4	2	0/Yes	0.55	−1.3	High	2.47
L25	77487	9	0	4	132.115	−0.13	4	1	0/Yes	0.85	−0.41	High	1.56
L26	10329	9	6	0	120.151	1.75	1	0	0/Yes	0.55	−2.43	High	1.49
L28	7464	10	6	1	135.209	2.46	0	1	0/Yes	0.55	−2.46	High	1
L37	500249	10	0	2	143.141	−0.55	3	1	0/Yes	0.55	−0.32	High	1.89
L38	412870	14	6	2	209.719	3.21	1	1	0/Yes	0.55	−3.4	High	2
L39	5012113	11	0	1	155.24	1.22	2	1	0/Yes	0.55	−1.44	High	2.65
L42	243165	11	6	1	153.141	−0.56	3	1	0/Yes	0.55	−1.57	High	2.05
L65	560622	20	0	6	282.378	1.66	4	0	0/Yes	0.55	−2.22	High	4.38

**Table 2:** Predicted toxicity profiles of selected compounds using ProTox 3.0 and DataWarrior.

S. No	Acute Oral Toxicity	LD50 of Rat (mg/kg)	Cardiotoxicity	AMES Toxicity	Carcinogenicity	Immunotoxicity	Cytotoxicity	Hepatotoxicity	Mutagenic	Tumourigenic	Reproductive Effective	Irritant
L3	4	1500	Inactive	Inactive	Active	Active	Inactive	Active	none	none	none	none
L4	5	2300	Inactive	Inactive	Active	Inactive	Active	Inactive	none	none	none	none
L6	4	1516	Inactive	Active	Active	Inactive	Inactive	Inactive	none	none	none	none
L9	3	360	Inactive	Inactive	Inactive	Inactive	Inactive	Inactive	none	none	none	none
L13	4	1600	Inactive	Inactive	Active	Inactive	Inactive	Inactive	none	none	none	none
L23	4	595	Active	Active	Inactive	Inactive	Inactive	Inactive	none	none	none	none
L24	3	550	Inactive	Inactive	Inactive	Inactive	Inactive	Inactive	none	none	none	none
L25	5	5000	Inactive	Inactive	Inactive	Inactive	Inactive	Inactive	none	none	none	none
L26	4	1743	Inactive	Inactive	Active	Inactive	Inactive	Inactive	none	none	none	none
L28	4	500	Inactive	Active	Active	Inactive	Inactive	Inactive	none	none	none	none
L37	4	1000	Inactive	Inactive	Inactive	Inactive	Inactive	Inactive	none	none	none	none
L38	4	450	Inactive	Inactive	Inactive	Inactive	Inactive	Inactive	none	none	none	none
L39	4	1000	Inactive	Inactive	Inactive	Inactive	Inactive	Inactive	none	none	none	none
L42	4	800	Inactive	Active	Active	Inactive	Inactive	Active	none	none	none	none
L65	3	180	Inactive	Inactive	Active	Active	Inactive	Inactive	none	none	none	none

**Fig 3:**
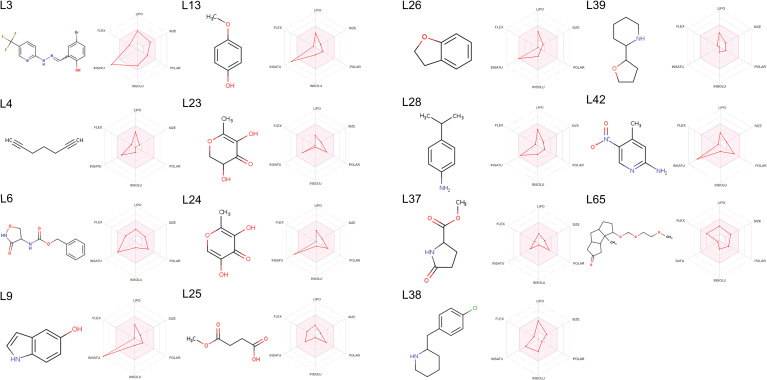
SwissADME radar plots.

SwissADME was also employed to graphically assess the drug-likeness of the 15 lead molecules through radar plots (Fig 3). In these plots, drug-likeness is depicted as a multi-parameter radar plot with each axis corresponding to a specific property, and the pink region marks the preferred range for those parameters. For the 15 leads, nearly all measured properties fell within this pink area, supporting their stability and potential as drug candidates [69].

The radar plots of 15 selected compounds lie predominantly within the coloured zone, indicating that they fall in the suitable physicochemical space for oral bioavailability, as defined by POLAR (Polarity), INSOLU (Insolubility), INSATU (Instauration), FLEX (Rotatable bond flexibility), LIPO (Lipophobicity), and SIZE (Molecular Weight).

### Protein physicochemical analyses

**Fig 4:**
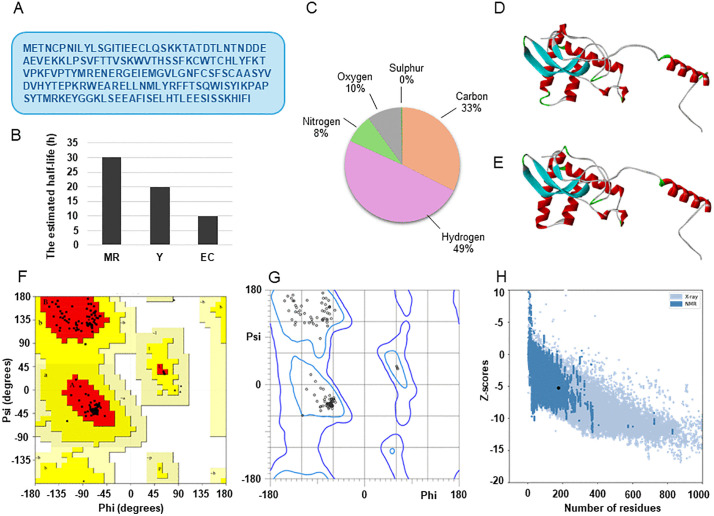
ASFV B175L physicochemical characterisation and modelling. (A) Amino acid sequence of ASFV B175L. (B) Predicted half-life of ASFV B175L in mammalian reticulocytes (MR), yeast (Y), and Escherichia coli (EC) (a, b, and c figures were generated based on details obtained from the ProtParam tool on the ExPASy server). (C) Atomic composition of B175L. (D) Discovery Studio visualised, the AlphaFold3 modelled ASFV B175L structure. (E) GalaxyWeb refined the structure of B175L, visualised using Discovery Studio. Ramachandran plot of the modelled and refined ASFV B175L obtained from (F) PROCHECK and (G) MolProbity. (H) ProSA Z score diagram.

As shown in Fig 4A the protein consists of 175 amino acids, with Glu (E) as the most abundant residue (19), followed by Ser (S, 17), Thr (T, 16), Leu (L, 13), Lys (K, 13), Ile (I, 10), Phe (F, 10), Tyr (Y, 9), Val (V, 9), Ala (A, 7), Asn (N, 7), Pro (P, 7), Arg (R, 6), Cys (C, 6), Gly (G, 6), His (H, 5), Met (M, 5), Asp (D, 4), Trp (W, 4), and Gln (Q, 2). In contrast, the rare residues Pyl (O) and Sec (U) were absent. The calculated molecular weight was 20,348.20 Da, and the theoretical isoelectric point (pI) was 5.77, indicating a moderately acidic protein with a slight excess of negatively charged residues (Asp + Glu = 23) over positively charged residues (Arg + Lys = 19), which may influence solubility and electrostatic interactions with ligands. The instability index (II) of 52.38 classified B175L as unstable, suggesting that the protein may be sensitive under *in vitro* conditions, whereas the aliphatic index of 70.17 indicates a considerable proportion of aliphatic side chains, consistent with reasonable thermostability. The GRAVY score reflects the global hydrophilic character and supports solubility. The GRAVY value of ASFV B175L was −0.355, supporting good solubility and favouring its use as a receptor in docking and dynamics simulations. The estimated half-life values were 30 h in mammalian reticulocytes, > 20 h in yeast, and >10 h in *Escherichia coli* (Fig 4B). These values further suggest the protein’s stability in heterologous expression systems, which is important for validating the *in silico* predictions. Finally, the molecular formula C_917_H_1395_N_229_O_273_S_11_ with a total of 2825 atoms (Fig 4C), provides a detailed compositional basis for future mass-spectrometric and structural studies [38].

### Protein structural modelling, refinement, and quality validation

We modelled the 3D structure of the B175L protein using AlphaFold3 (https://alphafoldserver.com/fold/25439d74b94763d2) (S1B Fig), which generated 5 high-quality models. Based on superior structural assessment scores, model 0 was selected for further studies (Fig 4D) (Table 3). The model provides insights into the protein’s overall fold and predicted structural characteristics. AlphaFold3 confidence metrics were used to assess the reliability of the generated structures. The selected AlphaFold3 model exhibited a pTM of 0.73, indicating a generally high level of global confidence in the overall structural prediction. According to the internal confidence assessment, the ranking score, which reflects AlphaFold3’s internal assessment of model quality, was 0.86 (>0.80), indicating that this model ranks among the top predictions for the B175L query. Analysis of the per-residue confidence profile revealed 26% of sequence regions predicted to be disordered, suggesting the presence of flexible regions within the B175L structure [70]. The model exhibited a ‘has clash’ value of 0.0, indicating that no significant steric clashes were detected in the predicted conformation. This supports the structural validity of the atomic geometry and suggests no major local packing, with no critical local packing defects at its prediction resolution [71]. As our analysis focused on the B175L protein in isolation, the model was generated without multiple chains. As a result, multimer-specific global interference metrics such as overall chain ipTM were null. The chain pair ipTM score was 0.73, along with the normalised chain pair PAE min of 0.76, indicating a moderately high level of confidence. Although these metrics are typically used for multimer models, in this monomeric context, they support arranging different regions of the B175L polypeptide in a consistent, geometrically reasonable manner within the overall structure, rather than representing an actual protein-protein interface used in multimer prediction [72].

**Table 3:** Structural validation metrics of the ASFV B175L model predicted by AlphaFold3, refined using GalaxyRefine, and evaluated using SAVES 6.0 and MolProbity web servers.

Tool/Server	Parameter	Value
AlphaFold3	pTM score	0.73
	Ranking score	0.86
	Fraction disordered	26%
	Chain pair ipTM	0.73
	Chain pair PAE min	0.76
GalaxyRefine	MolProbity score	1.256
	GDT-HA	0.9571
	RMSD	0.416 Å
	Clash score (per 1000 atoms)	4.9
	Poor rotamers	0.6%
SAVES 6.0	Ramachandran favoured	98.1%
	Ramachandran allowed	1.9%
	Ramachandran outliers	0%
	ERRAT	93.3333%
	VERIFY3D (≥0.1)	69.14%
MolProbity	Ramachandran favoured	100%
	Ramachandran outliers	0%
ProSA web	Z score	−5.26

The selected protein model's tertiary structure was refined using the GalaxyRefine server, which produced 5 refined models that optimised local structure through repeated side-chain packing and overall structural relaxation, thereby enhancing the model’s physical realism and increasing the number of residues in the favoured region. The changes in structure relative to the initial model are presented in a table. The test results in improvement of models over the initial input model for backbone structure accuracy by global distance test-high accuracy (GDT-HA), side chain structure accuracy measured by GDC-SC, and physical correctness measured by Molprobity score, number of atomic clashes per 1000 atoms by clash score, the percentage of rotamer outliers using poor rotamers, and Ramachandran favoured backbone torsion angles by Rama favoured [41]. The best refined model 4 was chosen for further studies based on combined refinement metrics: MolProbity score = 1.256, GDT-HA = 0.9571, RMSD = 0.416 Å, clash score = 4.9, and poor rotamers = 0.6% (Fig 4E).

The quality of the selected refined B175L model was comprehensively assessed using multiple independent tools (Table 3). Ramachandran plot analysis showed excellent stereochemical quality. PROCHECK reported 98.1% residues in the favoured region, 1.9% in the allowed region, and 0% outliers. MolProbity reported 100% residues in the favoured region with no stereochemical outliers, indicating a geometrically sound conformation of the predicted model (Figs 4F and 4G). The VERIFY3D score indicated that 69.14% of the residues have an average 3D-1D score ≥0.1, supporting acceptable agreement between the sequence and the local 3D environment. The ERRAT overall quality factor was 93.3333%, which falls within the range reported for reliable models. ProSA web server analysis yielded a Z score of −5.26, which falls within the distribution of high-quality experimentally determined structures of similar size, thereby reinforcing the validity of the predicted B175L fold (Fig 4H) [73,74].

### Computational docking and structural visualisation

Molecular docking and *in silico* analyses are essential tools widely used in modern drug discovery to estimate how small molecules fit within protein binding pockets, facilitating the identification of potential drug-like compounds based on binding affinity and interaction patterns [75]. In this study, docking analyses were used to evaluate the binding affinities of phytochemicals identified from *T. cordifolia* stem extract to the ASFV B175L protein, providing a preliminary basis for prioritising a subset of compounds for experimental validation. This scoring function estimates receptor-ligand stability by integrating key energetic terms (hydrogen bonding, electrostatic, van der Waals, and hydrophobic effects) to approximate binding affinity.

**Fig 5:**
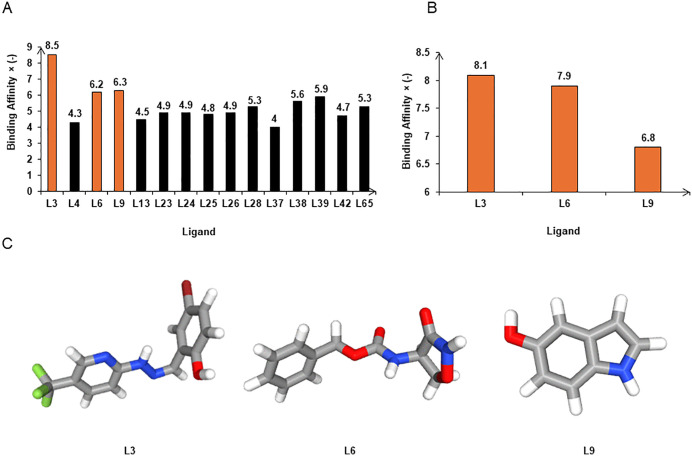
Graphical illustration of docking scores from PyRx virtual screening and CB-Dock2. (A) PyRx virtual screening results. This graph clearly illustrates docking scores for 15 ADME/T adhered compounds. Compounds with a binding affinity greater than −6 kcal/mol are represented by orange colour bars. (B) CB-Dock2 molecular docking results for the top 3 compounds. (C) Three-dimensional (3D) structures of the top 3 compounds from the PubChem database.

Through docking analysis, 15 ADME/T-compliant compounds (S1 File) were docked against the predicted B175L structure using PyRx 0.8 with AutoDock Vina Scoring (S2 File). To prioritise the most promising hits, a docking score threshold of ≤−6 kcal/mol was applied, as values below this level are commonly used in virtual screening studies to identify compounds with potentially stronger binding affinity [76–78]. The PyRx docking results ranged from −4 kcal/mol to −8.5 kcal/mol, and 3 compounds exceeded the cutoff binding energy at the active binding site (Figs 5A). The top 3 ligands, Benzaldehyde, 5-bromo-2-hydroxy-,(5-trifluoromethyl-2-pyridyl)hydrazone (L3), Carbamic acid, N-(3-oxo-4-isoxazolidinyl)-, benzyl ester (L6), and 1H-Indol-5-ol (L9) showed PyRx binding energies of −8.5, −6.2, and −6.3 kcal/mol, respectively. Analysis using CB-Dock2 server further validated these hits, as all 3 compounds showed strong binding affinities (L3: −8.1, L6: −7.9, and L9: −6.8 kcal/mol) (Fig 5B), they were comparable to the initial PyRx scores (Table 4). These findings effectively indicate high-affinity binding potential of the identified hits at target site.

**Table 4:** The binding affinities and non-covalent interactions of the top 3 selected compounds with ASFV B175L.

Compound	Docking Score (PyRx) (kcal/mol)	Docking Score (CB-Dock2) (kcal/mol)	Interacting Amino Acid Residues	Distance (Å)	Bond Category	Bond Type
L3	−8.5	−8.1	THR48	2.56965	Hydrogen	Conventional Hydrogen
			VAL46	2.45481	Hydrogen	Conventional Hydrogen
			PRO72	3.5427	Halogen	Halogen (Fluorine)
			PRO72	2.82304	Halogen	Halogen (Fluorine)
			PHE47	5.32418	Hydrophobic	Pi-Pi T-shaped
			VAL46	4.74154	Hydrophobic	Alkyl
			LEU43	4.13605	Hydrophobic	Alkyl
			PRO72	4.58338	Hydrophobic	Alkyl
			PHE74	4.86552	Hydrophobic	Pi-Alkyl
			PRO44	4.9547	Hydrophobic	Pi-Alkyl
			LYS52	5.35585	Hydrophobic	Pi-Alkyl
			PRO44	4.30411	Hydrophobic	Pi-Alkyl
			VAL54	5.24165	Hydrophobic	Pi-Alkyl
			VAL91	5.24664	Hydrophobic	Pi-Alkyl
L6	−6.2	−7.9	THR48	2.10141	Hydrogen	Conventional Hydrogen
			THR48	1.97177	Hydrogen	Conventional Hydrogen
			THR49	2.89911	Hydrogen	Conventional Hydrogen
			LEU43	4.32587	Hydrophobic	Pi-Alkyl
			PRO44	5.14309	Hydrophobic	Pi-Alkyl
			VAL54	4.92668	Hydrophobic	Pi-Alkyl
			PRO72	5.23855	Hydrophobic	Pi-Alkyl
			VAL91	5.16983	Hydrophobic	Pi-Alkyl
L9	−6.3	−6.8	PRO44	2.03927	Hydrogen	Conventional Hydrogen
			PRO72	1.83838	Hydrogen	Conventional Hydrogen
			VAL46	3.09211	Hydrogen	Carbon Hydrogen
			LEU43	5.44659	Hydrophobic	Pi-Alkyl
			PRO44	4.0523	Hydrophobic	Pi-Alkyl
			VAL91	4.72242	Hydrophobic	Pi-Alkyl
			LEU43	4.67596	Hydrophobic	Pi-Alkyl
			PRO44	5.42698	Hydrophobic	Pi-Alkyl
			VAL54	4.89082	Hydrophobic	Pi-Alkyl
			PRO72	5.14302	Hydrophobic	Pi-Alkyl

**Fig 6:**
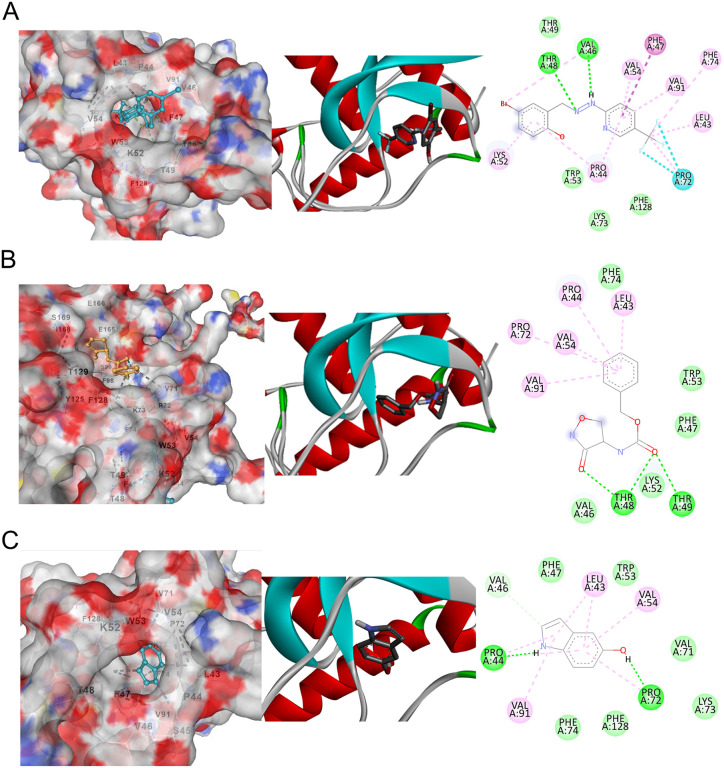
Molecular docking of ASFV B175L with the top 3 compounds. (A) L3, (B) L6, (C) L9 compounds docked in the ASFV B175L in 3D representation (left panels). The phytocompounds are shown in element-specific colours within the ribbon structure of ASFV B175L-ligand complexes at their docking poses (middle panel). The right panels show 2D interaction diagrams of 3 B175L-ligand complexes. Interaction in green colour indicates conventional hydrogen bonds, blue dotted lines indicate halogen interactions, dark pink dotted lines represent Pi-Pi T-shaped interactions, light green dotted lines for van der Waals interactions, and light pink lines indicate alkali and Pi-alkali interactions.

As shown in Table 4, L3 formed an extensive interaction network within the binding pocket. It interacted residues THR48, VAL46, LEU43, LYS52, VAL54, VAL91, PHE47, PHE74, PRO44, and PRO72, through a combination of 2 hydrogen bonds, 10 hydrophobic contacts (Pi-Alkyl, Alkyl, Pi-Pi T-shaped), and 2 halogen interactions, indicating strong complementarity with the local microenvironment. L6 established 3 hydrogen bonds and 5 hydrophobic interactions (Pi-Alkyl), engaging amino acid residues THR48, THR49, LEU43, PRO44, VAL54, PRO72, and VAL91. L9 formed 3 hydrogen bonds, 7 hydrophobic (Pi-Alkyl) interactions with residues PRO44, PRO72, VAL46, LEU43, VAL91, and VAL54, further supporting its capacity to stabilise within the same functional region (Fig 6).

ASFV B175L, an IFN antagonist that contains an MYM-type zinc finger with a conserved FSC motif, was identified as located approximately between residues 60–104 using NCBI conserved domain (S1C Fig). The clustering of contacts around residues within and immediately adjacent to the zinc finger region (60–104) was observed in L3, L9, and L6. The zinc finger region mediates interaction with STING and 2’3’-cGAMP and enables ASFV to suppress cGAS-STING-dependent antiviral signalling. Targeting this region is therefore a rational strategy to interfere with B175L function and to potentially restore host IFN response [17].

### MD simulations

To analyse the real-time dynamics and conformational stability of a protein bound to specific ligands, 100 ns of MD simulations were run for the top 3 receptor-ligand complexes [79,80]. The outcomes were compared across the complexes in terms of protein RMSD, ligand RMSD, protein RMSF, ligand RMSF, protein-ligand contacts, and ligand characteristics. These were evaluated based on the simulation interaction diagrams (SIDs) generated from trajectories of the 100 ns TIP3P water model [81].

To better interpret top-scoring ligand dynamics, a similar 100 ns MD simulation was performed for the negative-control complex, in which B175L was bound to the ligand with the weakest predicted binding affinity (L37). For this negative control, protein and ligand RMSD and RMSF profiles were evaluated to characterise dynamic behaviour. Specifically, RMSD and RMSF values from the negative control were compared to those from 3 selected B175L-ligand complexes (B175L-L3, B175L-L6, B175L-L9). The focus was on quantifying differences in both stability measures (RMSD, RMSF) to clarify how the dynamic stability contrasted with that of the top-scoring complexes.

### Root mean square deviation (RMSD) analyses

**Fig 7:**
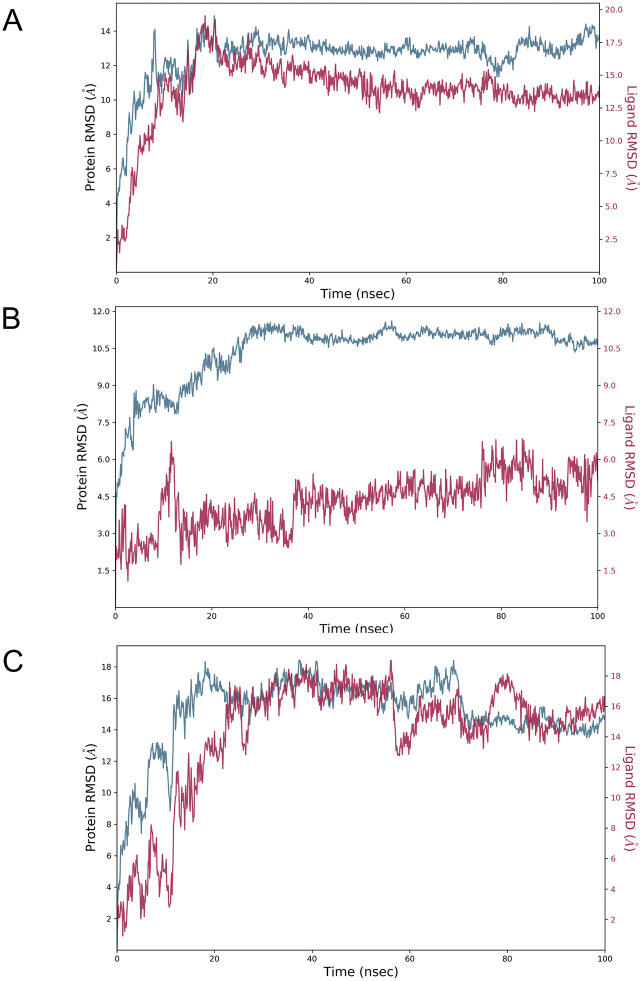
Protein backbone and bound ligand root mean square deviation (RMSD) profiles of the complexes during the molecular dynamics run. The charts show each complex's general structural stability and convergence tendency over time. (A) ASFV B175L-3L, (B) ASFV B175L-6L, and (C) ASFV B175L-9L.

We used RMSD to assess conformational stability of the B175L protein bound to the 3 top hit compounds [82]. For the B175L-bound compound L3 complex, initial fluctuations were observed in the first 0–20 ns, protein RMSD of 2–14 Å, and ligand RMSD stabilising in 2–13 Å. Convergence was achieved after 20 ns. Both protein and ligand maintained relatively stable dynamics, with protein RMSD of 11–14 Å, ligand RMSD of 10–13 Å throughout the remainder of the trajectory. Similarly, in the B175L-bound compound L9, both protein and ligand RMSD showed high initial fluctuations at 2–18 Å and 2–16 Å, respectively, over the first 0–20 ns of simulation. The values converged to higher but stable values after 20 ns. This suggests that the complex undergoes extensive early rearrangement before achieving stable dynamics post-equilibration. Thereafter, the RMSD values plateaued, with protein and ligand RMSD ranging from 14 to 18 Å and 13–16 Å, respectively, for the remainder of the simulation. In the B175L-L6 complex, protein RMSD fluctuated between 2–10 Å and ligand RMSD between 2–7 Å during the initial 0–20 ns. These values then remained converged throughout the simulation, with protein RMSD in the 9–11 Å range and ligand RMSD in the 5–7 Å range until the end of the trajectory. This reflects relatively lower equilibrium RMSD and improved conformational stability for B175L-L6 compared with other complexes. The 100 ns simulation showed that the compounds exhibit distinct initial RMSD fluctuations but reach stable plateau values beyond the 20 ns equilibration mark, with post-equilibrium fluctuation range remaining within a 4 Å range (Fig 7) [83].

For the negative-control B175L-L37 complex, the protein Cα RMSD increased gradually from about 2 Å to 17 Å during the initial 0–20 ns. It then fluctuated within a high plateau range of 9–16 Å for the remainder of the simulation. In contrast, the ligand RMSD remained relatively lower and stable at 3–8 Å for nearly half of the trajectory. This was followed by a sharp increase and large oscillations, reaching 54 Å and highly fluctuating between 50–70 ns, before settling to RMSD range of 18–23 Å at the end of the simulation. This pattern indicates that L37 initially samples a loosely confined pose, then undergoes considerable positional displacements. This is consistent with a highly flexible and weakly stabilised complex compared to B175L-L3, B175L-L6, and B175L-L9 (S2 Fig).

### Root mean square fluctuation (RMSF) analyses

**Fig 8:**
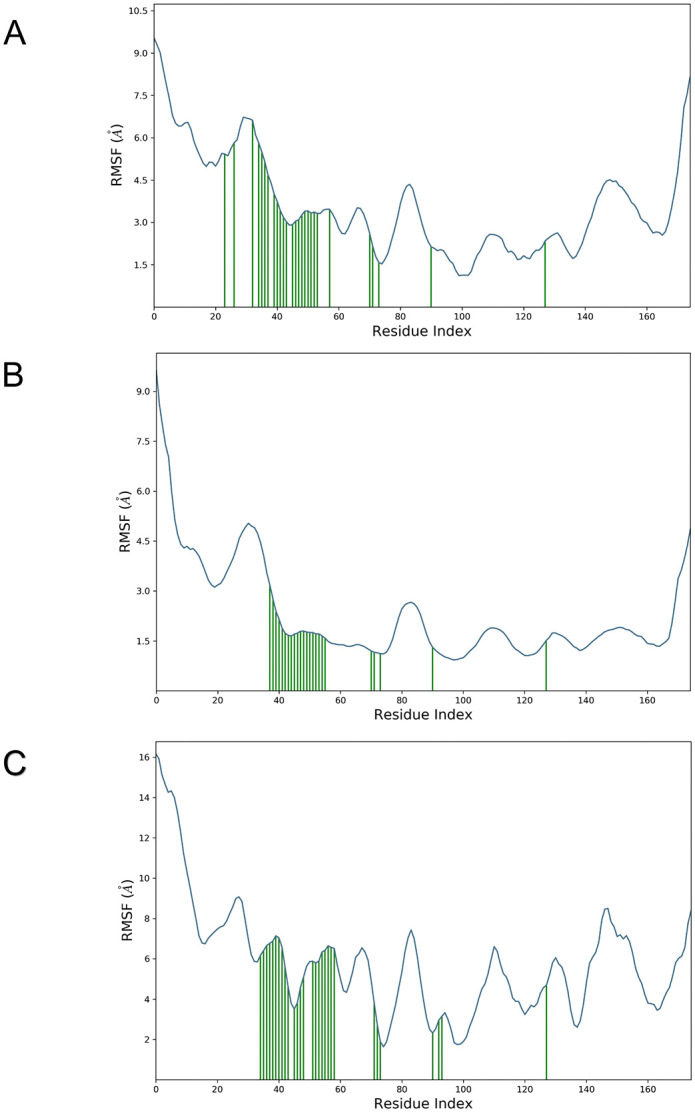
Root mean square fluctuation (RMSF) analyses of residue-wise flexibility of the protein chains in complexes. When the fluctuations of structured regions are lower, and those of loop regions are larger, the folding is stable with localised flexibility. (A) ASFV B175L-L3, (B) ASFV B175L-L6, and (C) ASFV B175L-L9.

**Fig 9:**
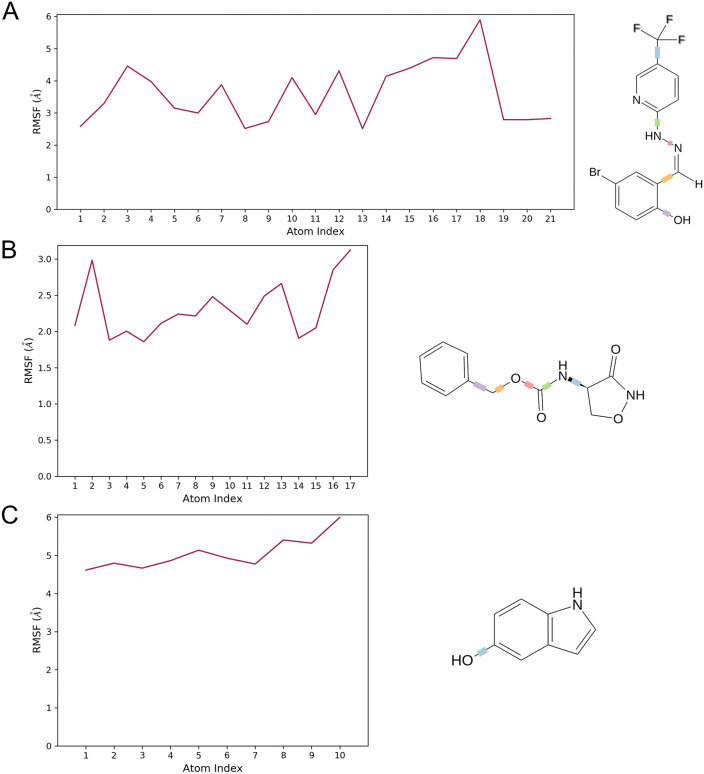
The flexibility of ligands complexed to ASFV B175L is represented by the root mean square fluctuation (RMSF) plots of ligands. All ligands remained stable in the binding pocket and maintained their conformations throughout the simulations, as evidenced by the modest fluctuation patterns. (A) ASFV B175L-L3, (B) ASFV B175L-L6, and (C) ASFV B175L-L9.

By calculating RMSF, we measured the thermodynamic flexibility and mobility of the B175L protein-ligand complexes [84]. As shown in Figs 8 and 9, elevated RMSF values indicate dynamic regions of the protein. Lower RMSF values suggest greater structural stability across the trajectory [82]. The protein’s backbone RMSF in the presence of compound L3 was 1.5–4.5 Å after 20 ns. The ligand RMSF profile showed its highest fluctuation at residue 18. For the B175L-L6 complex, protein RMSF residues mainly fluctuated in the range of 1.5–3.0 Å. This reflects restricted flexibility in the stable domain. The ligand RMSF reached its maximum at residue number 17. When binding compound L9, the protein backbone RMSF stabilised within 2.0–7.0 Å after the equilibration phase. No significant peak in protein RMSF corresponded to hydrogen bonding for the most flexible ligand residue 10. RMSF analyses of these 3 complexes showed that most backbone fluctuations are limited to flexible regions. The most mobile ligand atoms were not directly involved in persistent contacts or hydrogen bonding with the protein active site. This supports the overall stability and consistent interaction profiles of all 3 B175L-ligand binding events throughout the simulation period [83].

For the negative control B175L-L37 complex, the protein backbone RMSF profile showed elevated fluctuation. N-terminal amino acid residues reached values up to 18 Å. Mobility gradually decreased to 2–8 Å across much of the core region, then increased towards the C-terminus (S3 Fig). This widespread elevation in residue mobility indicates that L37 binding does not effectively restrict B175L backbone flexibility. In contrast, the L3, L6, and L9 complexes showed more compact RMSF ranges. The ligand RMSF for L37 showed the highest fluctuation at atom 5, and the overall ligand RMSF remained uniformly high, 15–16 Å across all ligand atoms. These results suggest that the L37 ligand does not adopt a well-stabilised pose and undergoes substantial internal motion throughout the trajectory (S4 Fig). Together, RMSF patterns suggest that the weakest affinity ligand forms a dynamically unstable complex, whereas L3, L6, and L9 promote more restrained backbone dynamics over the simulation period.

### Protein-ligand contacts

**Fig 10:**
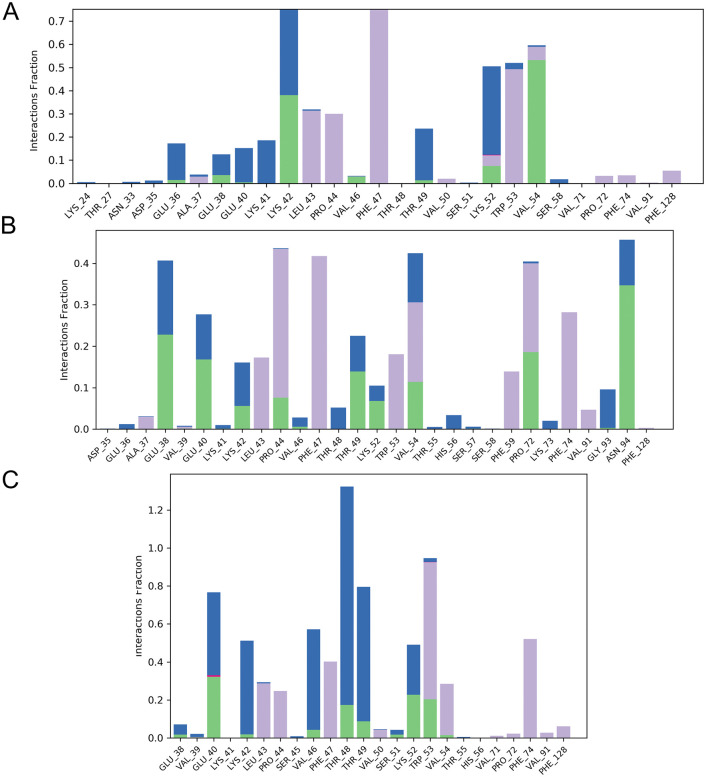
Protein ligand contact analyses indicate the number of intermolecular interactions that occur and how long they are in complexes between ASFV B175L and the ligands. The maintenance of hydrogen bonding (green), hydrophobic interactions (purple), and water bridges (blue) indicates stable and positive protein-ligand interactions. (A) ASFV B175L-L3, (B) ASFV B175L-L6, and (C) ASFV B175L-L9.

Detailed atomic-level interaction analyses were performed to predict the binding affinity between B175L and selected ligands during the MD simulations [85]. For compound L3, 24 amino acid residues in the protein were shown to interact with the ligand, forming hydrogen bonds, hydrophobic interactions, and water bridges throughout the 100 ns simulation period. In the case of compound L6, it exhibited interactions with 22 amino acid residues in the B175L binding pocket, characterised by hydrogen bonds, hydrophobic interactions, and water bridges. Similarly, compound L9, 26 amino acid residues engaged in significant contacts with the ligand, with observed interactions comprising hydrogen bonds, hydrophobic contacts, ionic bonds, and water bridges (Fig 10).

Most interactions involve the protein’s active-site residues. These interaction profiles collectively indicate that the extensive involvement of multiple B175L residues maintains a constant interaction with ligands, thereby prolonging the simulation period and supporting robust complex formation across all examined systems. Furthermore, protein-ligand interaction graphs revealed persistent hydrogen bonds and hydrophobic contacts throughout the simulations. The consistent engagement of multiple active-site residues indicates strong, sustained binding throughout the trajectory.

### Ligand root mean square deviation

**Fig 11:**
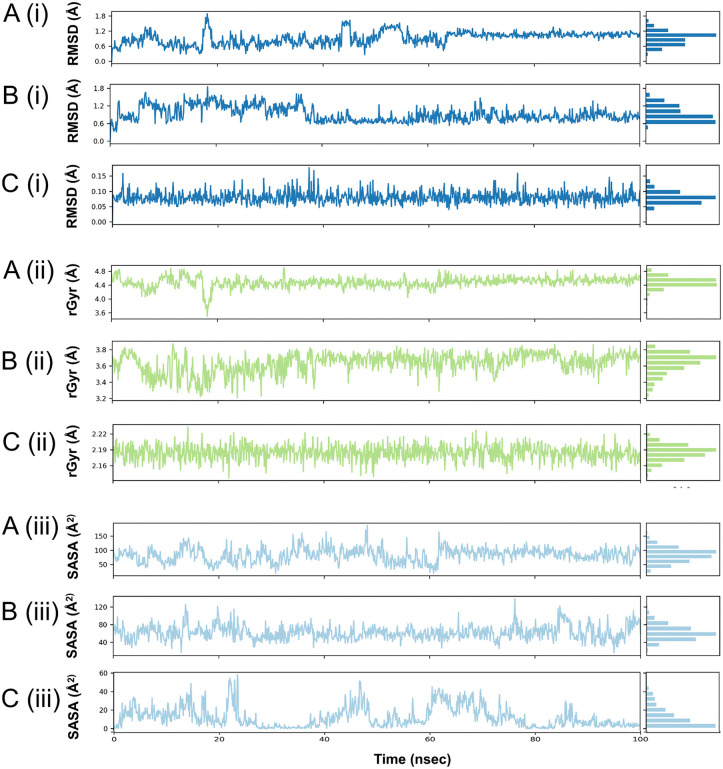
Changes in ligand’s root mean square deviation (RMSD), radius of gyration (rGyr), and solvent accessible surface area (SASA) during 100 ns simulations.

Root mean square deviation (RMSD) values for the 3 compounds were assessed throughout the simulation to assess their conformational changes upon binding to the B175L protein. Throughout the simulation, compound L9 remained below 0.15 Å, indicating a rigid and stable interaction in the binding pocket. Compounds L3 and L6 exhibited RMSD values in the range of 0.6–1.6 Å. Both L3 and L6 showed moderate initial fluctuations in RMSD, which stabilised near 1.2 Å after equilibration, reflecting stable compound conformations within the binding site. All compounds maintained RMSD values below 2 Å threshold, reflecting reliable structural stability during the simulation period (Fig 11) [82].

### Radius of gyration analyses

The radius of gyration (rGyr) determines the structural compactness and conformational behaviour of ligands during molecular dynamics simulations, reflecting the root mean square distance of the atoms from the centre of mass of the molecule [86]. The compound L3 rGyr, found to be in the range 4.2–4.8 Å, consistently remained below 5.0 Å throughout the trajectory, with only minor fluctuations, indicating a stable ligand configuration within the protein’s binding pocket and reflecting the system’s restricted flexibility. In the case of compound L6, a range of 3.3–3.8 Å was observed, indicating persistence, compactness, and stable interactions during the simulation period. Similarly, for ligand L9, the rGyr values were notably low, ranging from 1.8 to 2.2 Å, indicating a narrow range with no evidence of significant extension or unfolding (Fig 11).

### Solvent accessible surface area (SASA) analyses

The SASA quantifies the extent to which each compound surface is exposed to solvent molecules during the MD trajectory. Since biological machines function in an aqueous environment, it is essential for biomolecular modelling [87]. The compounds L3 and L9 ranged from 55 to 95 Å² and 40–70 Å², respectively, indicating the greatest degree of burial and intermediate solvent exposure. The compound L6 has a SASA of 80–120 Å², indicating that more of the molecule extends into the water (Fig 11).

Plots of ligand RMSD and rGyr of complexes with conformational stability and compactness of the ligands in the binding sites during the simulations. Ligand RMSD plots (Ai) ASFV B175L-L3, (Bi) ASFV B175L-L6, and (Ci) ASFV B175L-L9 for rGyr plots (Aii) ASFV B175L-L3, (Bii) ASFV B175L-L6, and (Cii) ASFV B175L-L9. The protein-ligand complexes have been investigated using SASA analyses, which show that the solvent surfaces are stable and that the binding environments do not change during the MD simulations. (Aiii) ASFV B175L-L3, (Biii) ASFV B175L-L6, and (Ciii) ASFV B175L-L9.

### MM-GBSA calculations

MM-GBSA analysis links the stability of each ligand within the binding pocket. It calculates the free energy of binding, which is responsible for chemical reactions, molecular recognition, association, and protein folding [88]. The results of MM-GBSA calculations are given in Table 5. Binding free energy calculations for the top 3 ligand-receptor complexes were performed using MM-GBSA based on stable trajectory segments from 100 ns MD simulations. The L3-B175L, L6-B175L, and L9-B175L complexes exhibited average binding free energies of −14.61, −12.81, and −9.21 kcal/mol, respectively, indicating formation of notably stable and energetically favourable complexes with B175L of L3 and L6 ligands compared to L9 [89].

**Table 5:** MM-GBSA binding free energy of the complexes using molecular dynamics trajectories.

Complex	ΔGbind	ΔGbind_Lipo	ΔGbind_Coulomb	ΔGbind_Hbond	ΔGbind_Packing
B175L-L3	−14.61	−5.95	−2.77	−0.14	−0.01
B175L-L6	−12.81	−5.92	−2.77	−0.06	−0.06
B175L-L9	−9.21	−3.51	−2.61	−0.09	−1.43

Estimates of MM-GBSA binding free energy of complexes based on MD trajectories. The findings provide an overview of total binding energies and the important energetic contributions to the favourable, stable binding of the ligands.

An overview of overall binding energies and significant energetic contributions to the ligands’ favourable and stable binding is given by the results in kcal/mol.

### Principal component analyses (PCA)

The MD trajectories were subjected to PCA to elucidate the key collective motions of the B175L in the presence of L3, L6, and L9. PCA projects the high-dimensional trajectory data set onto a low-dimensional space defined by the principal components (PCs), of which the first two (PC1 and PC2) capture the most significant scale variations of the system. The sampling of conformational stability and flexibility is represented by the distribution of trajectory points in the PC1, PC2 space of the protein-ligand complexes sampled in the simulation [90].

**Fig 12:**
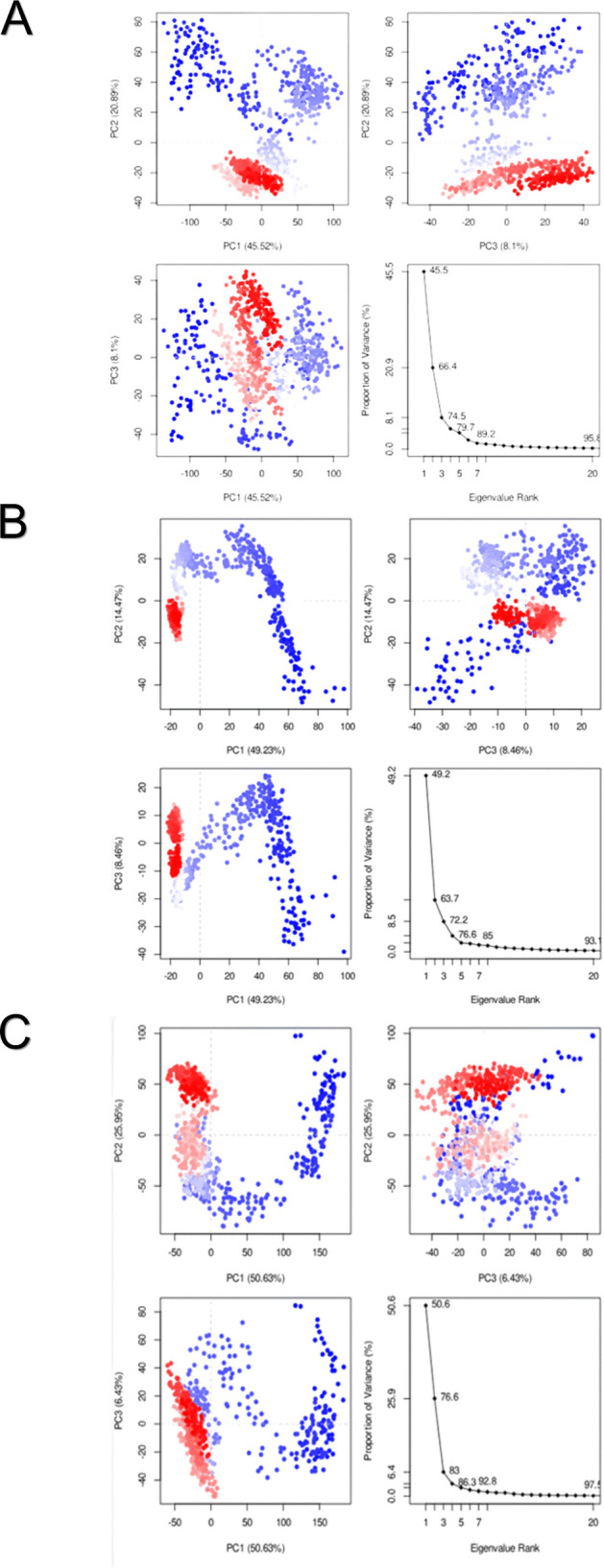
Principal component analyses (PCA) plots of the B175L-ligand complexes. PCA that captures the major common motions of protein-ligand complexes during molecular dynamics simulations. (A) B175L-L3, (B) B175L-L6, and (C) B175L-L9.

The B175L-L3 complex exhibited an intermediate distributional pattern compared to the other two complexes. Although the trajectory points were not very dispersed, extensions were observed mainly along PC1. This implies a trade-off: the system still possesses a degree of structural flexibility and is occasionally subject to the interchange of neighbouring conformational substrates. The L3 compound provides reasonable structural stabilisation by eliminating unnecessary conformational drift, but it is less restrictive than L9. The resultant distribution is consistent with ligands that make stabilising contacts while allowing some breathing of the structure and local movement (Fig 12A). On the other hand, the PCA plot of the B175L-L6 complex shows a broader, better-distributed distribution along PC1 and PC2. This trend indicates greater conformational flexibility and dynamic motion, and the system explored a significant number of microstates in the central component space. Such large dispersion implies that larger amplitude collective motions are available, and the ligand does not exert such strong structural restraint on the protein. The more conformational landscape suggests a more dynamic and flexible structure. It may well have been caused by weaker or reduced stabilising interactions between the ligand and the binding pocket, but this does not necessarily imply an unstable structure (Fig 12B). The B175L-L9 complex PC1PC2 scatter plot shows a very compact, tightly clustered distribution with low dispersion along the two major components. This limited clustering indicates that the protein-ligand complex experienced only a few large-scale collective motions and was trapped in a single, energetically stable conformational basin throughout the simulation. The lack of many clusters or long trajectories indicates that the binding of the ligands successfully limited the exploration of conformations and stabilised essential structural domains to a large extent. Such action is typical of a very stable complex, where variations are reduced mainly by the ligand’s stabilising effect (Fig 12C).

All the PCA results show that the protein’s dynamic behaviour and conformational sampling are directly regulated by the chemical nature of the bound ligand and the observed interaction profile. The B175L-L9 complex exhibited the highest conformational constraint, limiting the system to a single stable basin. The B175L-L6 complex exhibited the most significant conformational variation, enabling it to be more dynamic in its structural changes in exploring microstates. The stabilisation of the B175L-L3 provided intermediate stabilisation between coherence and localised flexibility. These results emphasise the significant effects of ligand-specific interactions on the protein’s functional movements and align with structural metrics such as RMSD, RMSF, and hydrogen-bond analyses.

## Discussion

Bioactive compounds from medicinal plants represent an important reservoir for new antivirals, and interest in their use has grown substantially in recent decades in response to emerging and remerging viral diseases. Flavonoids, alkaloids, terpenoids, and polyphenols, and other plant-derived metabolites have played a substantial role in modern antiviral drug discovery and continue to yield inhibitors with favourable safety and efficacy profiles against a wide range of human and animal viruses [91,92]. In particular, the unique structural and mechanistic diversity of phytochemicals enables them to modulate immune pathways and interfere with viral protein-host interactions [93]. *T. cordifolia* has a long-standing traditional, unique role in Ayurvedic medicine, particularly in the management of inflammatory and immune-related disorders, and its bioactive constituents have been reported to display notable immunomodulatory, antioxidant, and antiviral activities [94]. Experimental and clinical studies collectively suggest antiviral and immunomodulatory activity of *T. cordifolia* in the context of several RNA and DNA viral infections [94,95]. *In vitro* investigations have described inhibitory effects of *T. cordifolia* extracts against HAV, HIV, CHIKV RNA [12,16,96] viruses and HSV-1 DNA virus [14]. In addition, randomised clinical trials in patients with mild SARS-CoV-2 infection have shown that formulations containing *T. cordifolia* can accelerate viral clearance and improve inflammatory markers, supporting its use as an adjuvant antiviral therapy [15,97,98], and its immunomodulatory effects have also been documented in HIV-positive individuals and in preclinical models of viral infection [13]. This broad-spectrum of antiviral profile, combined with *T. cordifolia*’s capacity to modulate host immune responses, provides a rational basis for exploring its phytoconstituents as candidate inhibitors of viral infection. Its documented success in inhibiting DNA viral replication (e.g., HSV-1) suggests a potential mechanism of action that may extend to the ASFV.

African swine fever is a highly contagious and devastating viral disease that causes severe economic losses in the global swine industry. The absence of an effective antiviral drug or commercial vaccine makes disease management particularly challenging [99]. ASFV, the causative agent, exhibits complex transmission dynamics involving both mechanical and biological routes, which greatly complicates prevention efforts. Mechanical transmission occurs through contaminated feed, vehicles, meat, flies, and personal contact, while biological transmission involves infected pigs and soft ticks, further underscoring the difficulty of environmental control [100,101]. Upon infection, pigs exhibit a range of clinical signs such as vomiting, diarrhoea (sometimes bloody), internal bleeding, weakness, high fever, skin reddening, loss of appetite, abortion, and inability to stand, leading to a high mortality rate in acute cases [101,102]. Rapid detection and diagnosis of these clinical symptoms are critical for controlling outbreaks. The large double-stranded DNA genome further underscores its complexity, as it encodes numerous viral proteins that contribute to immune evasion and pathogenesis. These factors hinder efforts to develop effective treatments [103,104]. Among these proteins, the ASFV B175L protein has recently been identified as a reliable immune-evasion factor. It suppresses the type I interferon (IFN) signalling by targeting the STING adaptor complex, thereby suppressing the host’s innate antiviral response. This immunosuppressive function makes B175L an attractive target for antiviral intervention [17]. The complex immunoregulatory features and molecular biology of ASFV have led to growing interest in host-directed antiviral strategies that focus on modulating host signalling pathways and immune mediators as potential therapeutic approaches [105]. Although several small molecules and synthetic analogues have been investigated for their potential to inhibit viral immune evasion and host-virus interaction mechanisms, viral immune modulators remain relatively underexplored, highlighting the need for further experimental and translational efforts [106]. In this context, the present study used advanced computational methods to identify potential phytochemical inhibitors from *T. cordifolia* against ASFV B175L, offering a novel framework for therapeutic design against ASF. By integrating indigenous pharmacological knowledge with modern computational approaches, this research connects the Ayurvedic heritage of Sri Lankan medicine flora with contemporary antiviral drug design strategies aimed at mitigating ASFV pathogenesis.

This study identified 86 phytochemicals from *T. cordifolia* stem methanolic extracts by GC-MS, distributed across a variety of chemical classes, including fatty acids, esters, ethers and polyethylene glycols, macrocyclic compounds, crown ethers, alcohols, phenols, furanones, alkaloids, amines, nitriles, terpenoids, steroids and halogenated and miscellaneous organics such as halogenated esters, halogenated thioethers, sulfite esters, silylated derivatives (S1 Table). Methanol was used as an extraction solvent, which efficiently recovers antioxidant-rich fraction from plant material compared with several alternative solvents [107]. To ensure analytical reliability, phytochemical identification was validated by matching the acquired mass spectra against multiple reference libraries (NIST14 and Wiley9/NIST11 (W9N11)) [66], followed by peak deconvolution and probability-score filtering. Further enhancing confidence in the phytochemical assignments, GC-MS-identified phytochemicals were cross-checked in the PubChem database, and the corresponding compound curated structures and associated information were obtained and used for subsequent ADME/T and docking analyses [108,109]. To select lead compounds from this diverse chemical set, the identified compounds were subjected to pharmacokinetic and toxicological screening to identify those with an overall favourable balance of predicted efficacy and safety, thereby reducing the risk of later-stage failure [65]. 15 compounds satisfied the selection criteria. They showed favourable drug-likeness (no Lipinski’s rule violations and bioavailability ≥0.55) and acceptable solubility (LOG P, LOG S), and generally high predicted gastrointestinal absorption, consistent with their potential as oral drug candidates. Toxicity profiling indicated no predicted risks for mutagenicity, tumourigenicity, irritation, or reproductive toxicity. It classified these compounds in relatively favourable toxicity classes, with high lethal dose estimates, supporting their suitability for further development. This prioritisation allowed the retention of compounds with minor deviations in individual thresholds, provided their broader physicochemical, pharmacokinetic and safety profiles remained favourable.

Evaluation of physicochemical properties revealed that B175L is a moderately acidic, hydrophilic protein with favourable solubility, acceptable thermostability, and a predicted half-life compatible with heterologous expression, collectively supporting its use as a reliable receptor for structure-based docking, MD simulations, and future experimental validation [110,111].

In the absence of an experimentally resolved structure of B175L, an initial tertiary model was generated using the AlphaFold3 server, which has been shown to produce structures well-suited for virtual screening [110]. Among the predicted models, we selected model 0 based on a ranking score of 0.86, indicating high expected structural quality, and subsequently refined it to optimise local geometry. Validation analyses showed that nearly 98% of the amino acid residues fell within the favoured regions of the Ramachandran plot as assessed by PROCHECK and Molprobity, supporting good stereochemical quality. In addition, the ERRAT yielded an overall quality score of 93.33%, exceeding the commonly accepted threshold for high-quality comparative models [112]. ProSA-web returned a Z-score of −5.26, consistent with experimentally determined X-ray crystallography and nuclear magnetic resonance (NMR) structures of comparable size, and the residue-wise ProSA plot indicated a well-organised arrangement of amino acids, further supporting overall structural integrity [111].

Molecular docking was then employed to screen 15 ADME/T-compliant compounds against the modelled ASFV B175L structure. More negative docking scores were interpreted as indicative of stronger binding [110]. 3 ligand compounds (L3, L6 and L9) exhibited most favourable binding affinities (−6 kcal/mol or more) toward B175L, identifying them as the most promising phytochemicals for subsequent analyses [113]. A recent study reported comparable docking score ranges for *T. cordifolia* compounds against multiple viral targets, supporting the plausibility of these affinity values [114].

These ligands formed extensive noncovalent interaction networks within the predicted binding site, suggesting that their greater inhibitory potential reflects not only favourable binding energies but also strategic engagement of key local residues. The observed interaction pattern of B175L is consistent with the presence of a key contact residue within the pocket, where a limited number of interactions account for most of the stabilising energy. In contrast, other contacts contribute less to binding strength and specificity.

In the present study, all 3 ligands displayed the most extensive interaction profile, combining conventional hydrogen bonds, multiple hydrophobic contacts, and halogen interactions with B175L, which collectively enhanced their accommodation within the hydrophobic pocket. Hydrogen bonds assist the correct orientation of ligands within the binding site, whereas hydrophobic interactions strengthen binding affinity through favourable nonpolar interactions between protein and ligand [115]. Detailed mapping further revealed significant involvement of LEU43, PRO44, VAL54, PRO72, and VAL91 in ligand binding, indicating that these residues form an interaction hotspot on B175L, where hydrophobic interactions and hydrogen bonds synergistically enhance binding affinity and stabilise the complexes. Moreover, the clustering of contacts within and immediately adjacent to the B175L MYM-type zinc finger motif, which mediates binding to 2’3’-cGAMP and interaction with the cyclic dinucleotide-binding domain of STING, suggests that ligand engagement at this region may hinder proper assembly of the B175L-STING complex and disrupt the geometry required for efficient cGAMP engagement, thereby weakening B175L-mediated immune evasion. These observations agree with the previous studies of the B175L function [17].

The selected compounds, characterised by aromatic scaffolds and multiple heteroatoms, are efficient at forming hydrogen bonds, hydrophobic contacts, and halogen interactions, features that contribute to higher binding affinities and the selective protein-ligand recognition in the antiviral and other small-molecule therapeutics [116]. L3 incorporates a brominated phenyl ring and fluorinated pyridyl group, which can establish strong, directional contacts that can reinforce binding within a compatible pocket [117]. L9 features an indole core, a well-established antiviral chemotype, whose conjugated ring system and polar substituents support simultaneous π-staking and hydrogen bonding. At the same time, L6 combines a benzyl aromatic ring with a carbamate-linked heterocycle, providing a balance of hydrophobic surface and hydrogen-bond donor/acceptor groups. These features favour stable accommodation in protein grooves and are consistent with scaffolds used in modern small-molecule inhibitor design [118,119]. Molecular docking, although powerful, is limited by dependence on static receptor conformations and the possibility of inaccuracies in scoring. To address these limitations and refine the initial prediction, MD simulations were performed to assess the stability of the complexes over time [120].

All 3 ligand-protein complexes were subjected to 100 ns MD simulations. Although longer simulations can, in principle, capture additional conformational transitions, the system reached equilibrium within a 100-ns trajectory, sufficiently achieving the objective of identifying the most stable interactions. The chosen timescale aligns with previous MD studies evaluating the inhibitory potential of phytochemicals [47]. The B175L-ligand complexes exhibited distinct but convergent RMSD profiles, indicating that each system reached stable conformational dynamics after an initial equilibration period. Early fluctuations within the first 20 ns are likely to reflect structural adaptation, as commonly observed for protein-ligand systems [121]. Thereafter, RMSD values stabilised within ranges typical of a stable biomolecular system. These reduced deviations support a more rigid and tightly bound configuration, often associated with improved inhibitory potential [122]. RMSF analyses showed that ligand binding reduced the flexibility of key B175L residues, leading to lower backbone fluctuations and restricted movement within the binding pocket [123]. Elevated fluctuations were confined mainly to naturally flexible loop regions, whereas the active site remained comparatively stable. Such localised suppression of mobility upon ligand association is widely recognised as a key factor in achieving strong and stable binding. The MD simulation of the negative control ligand, which had the lowest binding affinity to B175L, further supported this trend. In comparison to the 3 top binding ligands, which showed higher binding affinities and multiple stabilising interactions, the lowest-affinity ligand (L37) produced less stable RMSD and RMSF profiles for both the protein and the ligand, indicating a more flexible and less well-stabilised complex. This contrast demonstrates that ligands with strong docking affinities are associated with greater dynamic stability, supporting the selection of L3, L6, and L9 as the more potent B175L binders. Ligand RMSD remained below 2 Å for all 3 compounds, indicating that ligands maintained their conformations within the B175L binding pocket [82]. Consistently, compact rGyr values for the ligands indicated restricted conformational flexibility and preserved structural integrity, which are typically associated with persistent binding [124]. In agreement with these findings, SASA analysis revealed limited solvent exposure of the complexes, suggesting stable, well-buried binding modes [125]. Among these 3 compounds, L9 displayed the lowest SASA and rGyr values, indicating a highly compact, reduced solvent-accessible nature, which is compatible with a strong, specific binding affinity [126].

The MM-GBSA analyses further indicated that all 3 ligands form thermodynamically favourable complexes with B175L. The more negative binding free energies for the calculated L3 and L6 suggest enhanced intermolecular complementarity, driven by persistent hydrogen bonding, hydrophobic interactions, and efficient packing with the active site [127]. These energetic results align with their stable MD behaviour, including favourable RMSD and RMSF profiles, compact ligand conformations, and sustained burial within the binding pocket, features commonly reported for promising small-molecule MM-GBSA-guided screening studies [63]. By contrast, L9 displayed a relatively higher ΔG, indicating lower stabilisation, despite its compactness and low SASA, and suggesting that the enthalpic contribution from specific contacts may be less optimal than for L3 and L6 [128]. PCA analyses offered additional insight into compound-induced dynamics and binding stability within the protein-ligand complexes. L9 and L3 showed a lower contribution of PC1 to the overall variance, indicating reduced larger-scale motions and greater stability in protein backbone dynamics relative to compound L6. After evaluating all these analyses, these findings indicate that the selected compounds emerged as potential candidates for achieving therapeutic effects in biological systems by targeting ASFV B175L.

The therapeutic efficacy of medicinal plants is closely linked to the diversity of their phytochemical profile. GC-MS, a widely used advanced analytical technique due to its high speed, accuracy, and sensitivity, enables the extensive detection of volatile and semi-volatile metabolites and provides insight into bioactive phytoconstituents [66]. However, several limitations should be considered when interpreting GC-MS profiles. This method is restricted to volatile, semi-volatile, and thermally stable compounds. Furthermore, extraction procedures, chromatographic conditions, and detector settings can affect quantitative detection and identification, sometimes leading to underrepresentation of low-abundance constituents. Electron-ionisation in GC-MS produces extensive fragmentation, which aids spectral library matching but can complicate interpretation for larger or novel molecules that are poorly represented in reference databases. Additionally, the high complexity of plant extracts can cause co-elution during GC separation, and the multiple steps of extraction, purification, and possible derivatisation are time-consuming and may result in the loss of volatile or unstable compounds. Consequently, complementing GC-MS-based profiling with LC-MS/MS or related liquid chromatography approaches can help overcome these limitations [129,130]. AlphaFold3 was used to generate high-quality computationally predicted protein structures when experimentally resolved models were unavailable [40]. However, docking against AI-modelled structures remains challenging. AlphaFold3 provides a static ground-state conformation, which may not fully capture local binding-site flexibility, side-chain rearrangements, or ligand-induced conformational changes that influence docking accuracy [131]. In addition, the lack of experimental cofactors in predicted models can impact the precise geometry of the binding pocket [132]. Systematic structural refinement and protein quality evaluation using multiple quality assessment tools improved stereochemical reliability prior to docking. Moreover, molecular dynamics simulations supported the complex’s stability under dynamic physiological conditions. Future studies employing experimental approaches, such as X-ray crystallography or NMR spectroscopy, will further refine AlphaFold3-derived models and thoroughly validate the predicted interactions [133,134]. Structural biology approaches have achieved great success in drug development for many diseases in the past, particularly by enabling structure-based screening of ligands against well-characterised targets [135]. They are especially valuable in antiviral research, where rapid viral emergence and resistance demand fast and rational drug discovery [136]. At the same time, *in silico* approaches become essential for accelerating early stages of drug discovery. However, computational methods cannot capture system-level biological functions such as off-target interactions, signalling networks, immune responses, toxicity, and whole-body pharmacokinetics [137,138]. In addition, most computational drug discovery models rely heavily on high-quality experimental data and are poorly generalisable to novel chemotypes, including structurally complex phytochemicals [139,140]. As a result, *in silico* techniques are valuable tools for early drug discovery in the antiviral pipeline [141,142]. To overcome these constraints, computationally prioritised compounds should be advanced into *in vitro* validation, including biochemical assays such as enzyme inhibition, surface plasmon resonance (SPR), isothermal titration calorimetry (ITC), and cell-based functional assays using cell lines, primary cells, or organoids [97,143]. Compounds that demonstrate antiviral activity *in vitro* could then be evaluated *in vivo* to characterise antiviral efficacy, pharmacokinetics, and toxicity in an integrated biological context [144]. In practice, the iterative design cycle of *in vitro* and *in vivo* results provides feedback to refine docking protocols, pharmacophore models, and machine learning predictions, reducing late-stage failures and bridging the gap between computational predictions and clinically effective drug discovery [142,145].

## Conclusion

This study integrated in-depth computational approaches to identify promising inhibitors from *Tinospora cordifolia* stem extract found in Sri Lanka, targeting the ASFV immune evasion protein B175L. A total of 86 phytochemicals were identified from methanolic stem extracts by GC-MS analysis and subsequently screened for pharmacokinetics, drug-likeness, and toxicity properties to select suitable drug candidates. In the absence of an experimentally resolved ASFV B175L structure, a high-quality 3D model was generated using AlphaFold3 and rigorously validated to ensure its suitability for molecular docking and molecular dynamics simulations. Docking as subsequent MD simulations highlighted 3 compounds (Benzaldehyde, 5-bromo-2-hydroxy-, (5-trifluoromethyl-2-pyridyl)hydrazone, Carbamic acid, N-(3-oxo-4-isoxazolidinyl)-, benzyl ester, and 1H-Indol-5-ol) that formed stable interactions with B175L over the simulation period, supported by binding energy analysis and principal component analysis, which together indicate their potential as ASFV B175L inhibitors derived from *T. cordifolia* stem. Nevertheless, these findings remain predictive, and further *in vitro* and *in vivo* studies are required to confirm their antiviral efficacy and assess their suitability for future therapeutic development against ASFV. However, also demonstrates the power of integrating traditional medicinal plant knowledge with modern computational drug discovery techniques.

## Supporting information

S1 Fig Graphical illustration of gas chromatography-mass spectrometry (GC-MS) chromatogram, modelled ASFV B175L, and zinc finger binding area of ASFV B175L. (A) GC-MS chromatogram of *Tinospora cordifolia* stem methanol extraction. (B) Modelled ASFV B175L structure using the Alphafold3 server. The model is coloured according to the predicted local distance difference test (pLDDT) score, a measure of the model’s confidence in the predicted structure. Regions coloured blue indicate very high confidence (pLDDT >90), cyan indicates confident regions (90 > pLDDT >70), yellow indicates low confidence (70 > pLDDT >50), and orange indicates very low confidence (pLDDT <50). (C) Zinc finger binding area of B175L, referring to NCBI conserved domain. The binding region of ASFV B175L is located between amino acid residues 60 and 104 within its 175 amino acid sequence.
(TIF)

S2 Fig Protein backbone and bound root mean square deviation (RMSD) profile of the negative control complex during molecular dynamics run. The chart shows general structural stability and convergence tendency of ASFV B175L-L37 over time.
(TIF)

S3 Fig Root mean square fluctuation (RMSF) analysis of residue-wise flexibility of the protein chains in ASFV negative control complex. Per-residue RMSF values of Cα atoms are shown along the sequence of ASFV B175L for the L37-bound system.
(TIF)

S4 Fig The flexibility of the L37 ligand complexed to ASFV B175L is represented by the root mean square fluctuation (RMSF) plot of the ligand. The RMSF values for individual heavy atoms of L37 are plotted to intra-ligand flexibility inside the binding pocket.
(TIF)

S1 Table List of compounds identified in the GC-MS analysis of *Tinospora cordifolia* stem methanol extract. (XLSX)

S2 Table Predicted pharmacokinetic properties and toxicity profiles of compounds identified in the GC-MS analysis of *Tinospora cordifolia* stem methanol extract using SwissADME, ProTox 3.0, and Datawarrior. (XLSX)

S1 File Three-dimensional SDF files of ADME/T approved plant compounds in PDB format. (ZIP)

S2 File Energy minimised (Universal Force Field (UFF)) ligand structures in PDB format. (ZIP)
