# Correction: *In silico* pharmacological analysis of *Tinospora cordifolia* compounds targeting African swine fever virus B175L

**DOI:** 10.1371/journal.pone.0356732

**Published:** 2026-08-20

**Authors:** Kasuni Karunarathne, Saumya Poorni, Nethra Jayampathi, Hasitha Dhananjaya, Anju Hasintha, Malinda Hulugalla, Thilini A. N. Mahakapuge, Nadeeka Nethmini, Naveed Iqbal, Barana Jayawardana, Lakmal Ranathunga

The third author, Nethra Jayampathi, should not have been attributed equal contribution to this work.

There are errors in the Funding statement. The correct Funding statement is as follows: This work was supported by the University Research Council (URC) Multidisciplinary Research Grant (No. 516), University of Peradeniya, awarded to LR, MH, TANM, and NN. The funder provided support in the form of research materials and salary support for author SP, but did not have any additional role in the study design, data collection and analysis, decision to publish, or preparation of the manuscript.
